# The Effects of Bladder Diverticulum on the Pathophysiology of Bladder Outlet Obstruction: An Experimental Study in Guinea Pigs

**DOI:** 10.14744/SEMB.2020.45389

**Published:** 2021-12-29

**Authors:** Aslihan Karadag, Suleyman Celebi, Feryal Gun Soysal, Ipek Yildiz Ozaydin, Erbug Keskin

**Affiliations:** 1. Department of Pediatric Surgery, Istanbul University Faculty of Medicine, Istanbul, Turkey; 2. Department of Pediatric Surgery, Division of Pediatric Urology, Kanuni Sultan Suleyman Training and Research Hospital, Istanbul, Turkey; 3. Department of Pathology, Kanuni Sultan Suleyman Training and Research Hospital, Istanbul, Turkey; 4. Department of Pediatric Surgery, Division of Pediatric Urology, Istanbul University Faculty of Medicine, Istanbul, Turkey

**Keywords:** Bladder diverticulum, partial bladder outlet obstruction urodynamic study

## Abstract

**Objectives:**

This study is the first to urodynamically and histopathologically evaluates the effects of bladder diverticulum (BD) secondary to bladder outlet obstruction (BOO).

**Methods:**

Guinea pigs (n=32) weighing 900–1,000 g were divided randomly into four groups: Sham, BD, BOO, and BD combined with BOO. All guinea pigs in the four groups underwent urodynamic evaluation preoperatively and at 1 month postoperatively. The bladders were removed and examined histopathologically.

**Results:**

The post-operative filling detrusor pressure was lower in the Sham group (7.1±1.6 cm H_2_O) than in the BD (21.4±5.6 cm H_2_O) and BD with BOO groups (23.6±9.3 cm H_2_O) (p<0.05). There was no difference between the Sham and BOO (9.5±4.0) groups. Post-operative bladder compliance was better in the Sham group (2.3±0.8 ml/cm H_2_O) than in the BD (0.9±0.22 ml/cm H_2_O) and BD with BOO groups (0.6±0.3 ml/cm H_2_O) (p<0.05). Involuntary detrusor contraction was not observed in the Sham or BOO groups, but was observed in 37.5% of subjects in the BD and BD with BOO groups. On histological examination, the bladder wall was thicker (3.75±0.68 mm) (p=0.601), and the connective tissue volume was significant increased (p=0.046), in the bladder muscularis mucosa in the BD groups compared to the BOO group.

**Conclusion:**

Physiological and histopathological changes in the bladder with BD combined with BOO are more evident than with BOO alone.

To maintain normal function, the highly specialized bladder wall must remain compliant.^[1,2]^ During bladder filling at physiologic rates, the detrusor pressure remains nearly constant because of a special property of the bladder known as accommodation.^[3]^ Accommodation is affected by the viscoelastic properties of the bladder, based on its composition of smooth muscle, collagen, and elastin.^[4]^

Regardless of the etiology, bladder outlet obstruction (BOO) leads to compression of, or resistance in, the bladder outflow channel; this can occur at any point from the bladder neck to the urethral meatus. This induced resistance initiates a pathophysiologic bladder response. One resulting pathology is bladder diverticulum (BD). BD is characterized by a thin bladder wall with noncontractile outpouching, which can lead to urinary accumulation, micturition problems, urinary incontinence, urolithiasis, and infection.^[3,4]^ At present, little is known about the diagnosis and treatment of BD, and there is no report in the literature describing the efficacy of BD combined with BOO.

The aim of this experimental study was to examine bladder physiology taking urodynamics into account, as well as bladder filling and voiding characteristics, contractile dysfunction, simultaneous pressure/flow analysis, and their associations with pathological examination results in a guinea pig model of BD with BOO.

## Methods

This study was conducted between December 2016 and December 2017 following approval by the Istanbul Medicine University Animal Experimentation Ethics Committee (no. 2016/70). Thirty-two guinea pigs weighing 900–1000 g each were divided randomly into four groups of eight animals each: The sham group, BD group, BOO, and BD with BOO group.

Urodynamic study (UDS) was performed on animals in all four groups without pre-operative anesthesia or sedation. After completely emptying the bladder, a 6F transurethral urodynamic catheter was introduced to the bladder and a rectal catheter was introduced 2 cm beyond the anal margin. The bladder was filled with saline at an infusion rate of 2 mL/min. Pressure-flow measurement and recording were performed with a videourodynamics system (Aymed Locum, Aymed Medical Technology, Istanbul, Turkey). UDSs were repeated 3 times and the averaged urodynamic findings were recorded. Cystometric bladder capacity (BC) was defined as the infused saline volume before urination, and bladder compliance was calculated as the change in volume divided by the change in pressure. Filling detrusor pressure (FPdet), and maximum voiding pressure (VPdet) were measured. Uninhibited contractions were considered as those detrusor involuntary contractions with low vesical volume, regardless of whether they yielded simultaneous urinary leakage.

**Figure 1.**
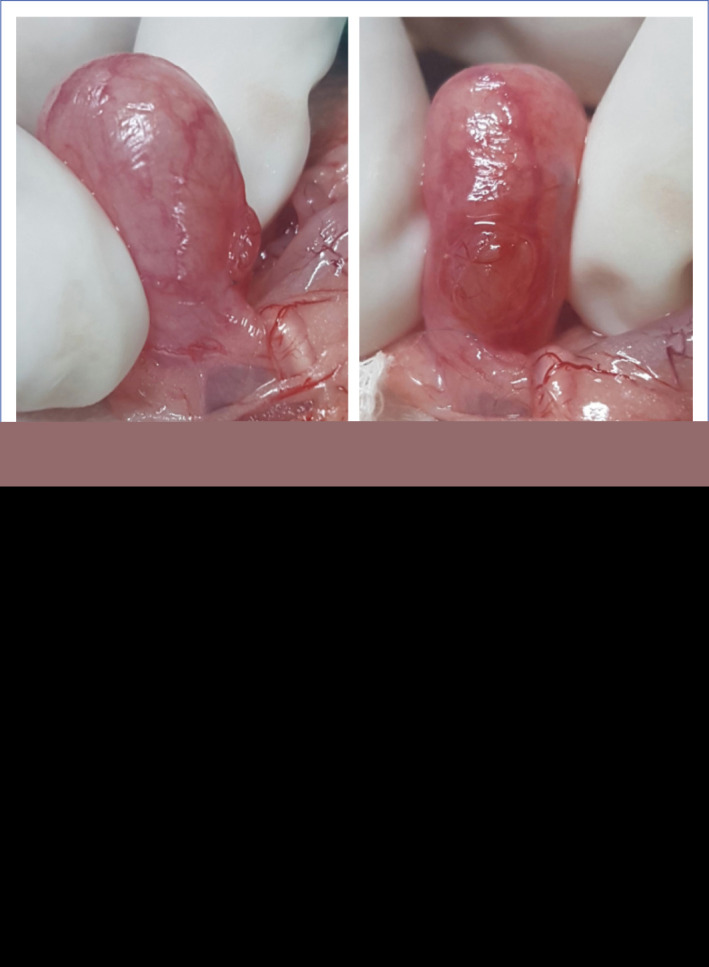
Bladder Diverticulum model.

**Figure 2.**
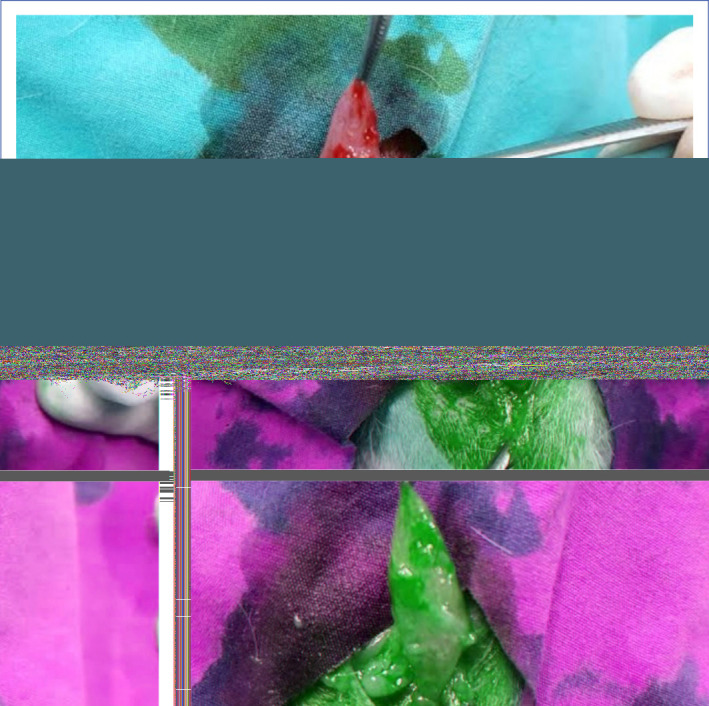
Partial bladder outlet obstruction model.

After pre-operative urodynamic testing, a 6-French (Fr) ureteral catheter was inserted from the urethra of each animal. Under anesthesia with ketamine and xylazine, a 3-cm median incision was made in the anterior abdominal wall. The ureters, bladder, and urethra were exposed by dissection of the surrounding tissues. For Sham group, the anterior abdominal wall and skin were closed without further modification. For BD group, following the procedure outlined above, the detrusor muscle fibers were partially excised to prevent reunion of the two sides over the ureter. The bladder mucosa was then prolapsed between the detrusor muscle fibers. Care was taken to maintain mucosal integrity. As even a large diverticulum will have a small neck, the entrance hole of the diverticulum was reduced by applying a 5/0 suture from the detrusor layer surrounding the diverticulum (not from the mucosal layer). The neck diameter was reduced to a size to pass an 6F urethral catheter (Fig. 1).^[3]^ For group 3, a BOO model was induced: A fascial band was removed from the anterior rectus fascia. The removed fascial strip was passed under the urethra inside the 6-Fr urethral catheter, and the urethra was surrounded and enclosed using the fascial strip and sutured with 4/0 Vicryl (Fig. 2).^[5]^ For group 4, a BD with BOO were induced simultaneously using the procedures outlined above for groups BD and BOO groups.^[4-6]^

**Figure 3.**
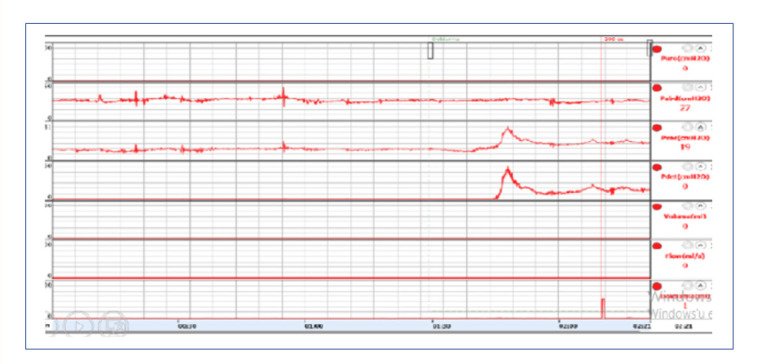
Urodynamic testing was performed on animals in Sham group. Postoperative filling detrusor pressure values were normal range in sham group and involuntary detrusor contraction was not observed.

At 1 month postoperatively, all subjects underwent urodynamic testing again (Fig. 3), and cystectomy was then performed under general anesthesia. Tissue samples were collected onto slides (4 μm thick) using a microtome (RM2255; Leica), and then deparaffinized. After staining with hematoxylin and eosin, the slides were examined under a light microscope (BX51; Olympus) to determine the presence of diverticula, the thickest and thinnest muscle layers on the bladder wall and the increase in connective tissue in the muscle layer. The increase in connective tissue was classified as mild (+1), moderate (+2), or strong (+3).^[7]^

The SPSS 22.0 Statistics program was used for statistical analysis. Pearson’s Chi-squared test was used to compare categorical data between groups. The Kruskal–Wallis test was used to compare the other measurement parameters between groups. The Wilcoxon test was used to examine the significance of intra-group differences between preoperative and postoperative values. The results were evaluated at a 95% confidence interval and p<0.05 significance level.

## Results

Post-operative FPdet was lower in the Sham group (7.1±1.6 cm H_2_O) than in BD (21.4±5.6 cm H_2_O) and BD with BOO groups (23.6±9.3 cm H_2_O) (p<0.05), but was not different between the Sham and BOO groups (9.5±4.0). In groups with BD, FPdet was significantly increased postoperatively relative to the pre-operative values (p=0.013) (Table 1).

**Table 1.** Comparison post-operative late periods of the Sham, BD and BOO groups

	**Preoperative values**	**Postoperative Sham**	**Postoperative BD**	**Postoperative BOO**	**Postoperative BD with BOO**
FPdet	6.1±1	7.1±1.6	21.4±5.6	9.5±4	23.6±9.3
VPdet	40±5	38.5±12	43.6±22.3	52±35.3	55.3±19.3
Compliance (cm H_2_0/ml)	2.1±0.5	2.3±0.8	0.6±0.3	1.3±0.5	0.9±0.3
Detrusor thickness (mm)	2.5±0.53	3.05±1.16	3.1±0.76	3.75±0.68
IDC (n)	0	0	3	0	3
Capacity (ml)	15±2	13.4±1.8	15±4.1	16.5±1.9	16.6±2.7
Connective tissue (n) (range 0–3)				
no		0	2	0	0
+1		4	4	4	2
+2		2	2	2	4
+3		1	2	0	2

VPdet: Maximum voiding pressure; FPdet: Filling detrusor pressure; BD: Bladder diverticulum; BOO: Bladder outlet obstruction.

Although the largest voiding pressure increase compared to the preoperative values was seen in the BD with BOO group (from 40±5 to 55.3±19.3 cm H_2_O), no significant difference between pre-operative and post-operative VPdet values was detected in any group (p=0.916).

Post-operative bladder compliance levels were higher in the Sham group (2.3±0.8 ml/cm H_2_O) than in the BD (0.6±0.3 ml/cm H_2_O) and BD with BOO groups (0.9±0.3 ml/cm H_2_O) (p<0.05), with no significant difference between the Sham and BOO (1.3±0.5 ml/cm H_2_O) operation groups (p>0.05).

No significant difference in post-operative BC was found among the groups (Table 1) (p=0.086). Involuntary detrusor contraction (IDC) was not observed in the sham or obstruction operation groups, but was observed in 37.5% of animals in the BD and BD with BOO groups.

Histopathologically, although the thickness of the bladder wall was greatest in the BD with BOO group (3.75±0.68 mm), no significant difference was found among the groups (p=0.601). However, there was a significant increase in connective tissue volume in the muscularis mucosa in the BD with BOO group (χ^2^=10.057, p=0.046).

## Discussion

Animal models of BOO show structural, functional and molecular changes similar to those in humans and have improved our understanding of many aspects of the pathophysiology of the condition.^[7,8]^

BOO has a variety of etiologies, which may be functional or anatomic, and leads to voiding dysfunction. Symptoms such as irritative voiding with a reduced flow rate, reduced or increased micturition pressure, increased duration of micturition, and incomplete emptying with increased residual volume are signs of obstructive dysfunction,^[9,10]^ and lead to smooth muscle hypertrophy and the accumulation of collagen and elastin in connective tissue.

Over time, to overcome the resistance of the obstruction, the intravesical pressure increases to 2–4 times the normal level in response to hypertrophic detrusor contraction, pushes the bladder mucosa out between the trabeculations and causes comorbidities such as the small pouches known as BD.^[11,12]^ BD can lead to urgency, frequency, and nocturia. As the BD wall has no muscle layer, the contents are not discharged and chronic infection will develop, even if the obstruction is removed.^[13]^

UDS provide critical data for management and treatment planning, and represent an integral component of assessments of complicated BOO. We used UDS to determine if BD is the cause or result of bladder dysfunction.^[14,15]^ BOO caused major changes in voiding physiology, but most of the main changes are due to the association with BD. Voiding pressure was increased slightly and compliance was dramatically lowered. Such reduced compliance is caused by a rapid increase in the filling phase. We correlated these findings with the pathophysiological results. Filling and voiding impairment resulted in increased bladder wall thickness and connective tissue volume.

It is ^[16]^ reported that the bladder wall was 4 times thicker in patients with BOO than in those with no obstruction. In our study, bladder wall thickness and connective tissue volume increased in all groups, with the largest increase being seen in the BD with BOO group. Connective tissue and muscular hypertrophy were most abundant around the diverticulum, and thickened fibrosis was present in the outer part of the diverticulum. This guinea pig study indicated that BD could further reduce the compliance of the bladder after BOO. Furthermore, the decrease in compliance correlates with progressive decompensation of the bladder.

In our study, as expected, BOO filling pressures (9.5 cm H_2_O) increased compared to the sham (6.1 cm H_2_O) group and likewise caused low compliance (1.3:2.1). This was confirmed in histopathological results. However, the incredibly strong response of the bladder detrusor to compliance prevented this pathology from being seen statistically significant. However, we observed that even relatively short post-operative period when a pathology that disrupted the detrusor muscle integrity was added to the bladder like BD, the compliance was extremely reduced (0.9:2.1) and became statistically significant.

This study was inspired by an experimental model that we described earlier.^[5]^ The most important limitation of this study was the requirement for bladder exploration and surgery to create BD and BOO. However, the BDs were created using a method that kept the uroepithelium intact. Therefore, we believe that the observed urodynamic changes were due to the presence of BOO and BD, and their negative effects on filling pressures. Further studies with longer follow-up in larger experimental animals or an artificial bladder created with BD, BOO are required to fully assess the pathophysiology of BOO and BD.

## Conclusion

This study is the first to evaluate the utility of a BD with BOO model for exploring the voiding physiology. The findings indicated that internal bladder dynamics are affected by events such as BD, rather than by obstruction alone. The model assumes that such events are secondary to BOO and contribute to a poor prognosis.

### Disclosures

**Ethics Committee Approval:** Istanbul University Medicine Faculty (2016/70).

**Peer-review:** Externally peer-reviewed.

**Conflict of Interest:** None declared.

**Authorship Contributions:** Concept – A.K., S.C.; Design – A.K., S.C.; I.O., F.G.S., E.K.; Supervision – A.K., S.C.; I.O., F.G.S., E.K.; Materials – A.K., S.C.; I.O., F.G.S.; Data collection &/or processing – A.K., S.C.; Analysis and/or interpretation – A.K., S.C., E.K.; Literature search – A.K., S.C., E.K.; Writing – A.K., S.C., I.O.; Critical review – S.C., A.K., E.K.
